# Japanese Knotweed Rhizome Bark Extract Inhibits Live SARS-CoV-2 In Vitro

**DOI:** 10.3390/bioengineering9090429

**Published:** 2022-09-01

**Authors:** Urška Jug, Katerina Naumoska, Tadej Malovrh

**Affiliations:** 1 Laboratory for Food Chemistry, Department of Analytical Chemistry, National Institute of Chemistry, Hajdrihova 19, 1000 Ljubljana, Slovenia; 2 Veterinary Faculty, University of Ljubljana, Gerbičeva ulica 60, 1000 Ljubljana, Slovenia

**Keywords:** Japanese knotweed rhizome bark, SARS-CoV-2, COVID-19, virus neutralization test

## Abstract

Coronavirus disease 2019 (COVID-19), a viral infectious respiratory disease, is caused by highly contagious severe acute respiratory syndrome coronavirus 2 (SARS-CoV-2) and is responsible for the ongoing COVID-19 pandemic. Since very few drugs are known to be effective against SARS-CoV-2, there is a general need for new therapeutics, including plant-based drugs, for the prophylaxis and treatment of infections. In the current study, the activity of a 70% ethanolic_(aq)_ extract of the rhizome bark of Japanese knotweed, an invasive alien plant species, was tested for the first time against the wild-type SARS-CoV-2 virus using a specific and robust virus neutralization test (VNT) on Vero-E6 cells, which best mimics the mechanism of real virus–host interaction. A statistically significant antiviral effect against SARS-CoV-2 (*p*-value < 0.05) was observed for the 50.8 µg mL^−1^ extract solution in cell medium. A suitable extract preparation was described to avoid loss of polyphenols throughout filtration of the extract, which was dissolved in cell medium containing fetal bovine serum (FBS). The significance of the differences between the sums of the test and control groups in the incidence of cytopathic effects (CPE) was determined using the one-way ANOVA test. A dose–response relationship was observed, with the cytotoxic effect occurring at higher concentrations of the extract (≥101.6 µg mL^−1^). The obtained results suggest possible use of this plant material for the production of various products (e.g., packaging, hygiene products, biodisinfectants, etc.) that would be useful against the spread of and for self-protection against COVID-19.

## 1. Introduction

Severe acute respiratory syndrome coronavirus 2 (SARS-CoV-2) [1] is a virus that is contagious to humans and some animal species and causes coronavirus disease 2019 (COVID-19), a viral respiratory illness responsible for the ongoing COVID-19 pandemic [2] declared by the World Health Organization on 11 March 2020 [3]. SARS-CoV-2 belongs to the *Coronaviridae* family, represented by enveloped viruses containing a positive-sense, single-stranded ribonucleic acid (+ssRNA) [4,5,6]. SARS-CoV-2 and other coronaviruses have four structural proteins known as spike, envelope, membrane, and nucleocapsid proteins. During viral infection, the spike protein promotes binding and fusion between the virus and the cell membrane [7]. The virus invades human cells by binding to the cell surface receptor angiotensin converting enzyme 2 (ACE2), a membrane glycoprotein [8,9]. The virus is transmitted from person to person mainly by close contact via aerosols and droplets from the respiratory air [10]. The nasal cavity respiratory epithelium is probably the predominant site of initial infection and virus replication [11]. Common symptoms and signs of a person infected with coronavirus include fever, cough, dyspnea, lymphopenia, abnormal results of chest computed tomography (CT), myalgia or fatigue, sputum production, headache, hemoptysis, diarrhea, etc. In more severe cases, the infection leads to acute respiratory distress syndrome, RNAemia, cardiac damage, secondary infections, and also death [12].

Depending on the target, there are two potential approaches to anti-coronavirus therapy, one targeting the coronavirus itself and the other targeting the support of the human immune system or human cells [7]. To date, few drugs are known to effectively inhibit SARS-CoV-2. Among specific drugs against SARS-CoV-2, Veklury (remdesivir) and Olumiant (baricitinib) have been approved by the FDA [13]. The EMA has approved several treatments for COVID-19: Evusheld (tixagevimab/cilgavimab), Kineret (anakinra), Paxlovid (PF-07321332/ritonavir), Regkirona (regdanvimab), RoActemra (tocilizumab), Ronapreve (casirivimab/imdevimab), Veklury (remdesivir), and Xevudy (sotrovimab) [14].

Plant-based drugs, including Japanese knotweed, have been used to treat various viral infections. The identification of phytochemicals with health-beneficial effects would be crucial for the development of new drugs against SARS-CoV-2 infection.

Japanese knotweed (*Fallopia japonica* Houtt., *Reynoutria japonica* Houtt., *Polygonum cuspidatum* Siebold & Zucc.) is an alien plant species native to East Asia that has become invasive in Europe and North America [15]. The rhizome extracts of Japanese knotweed have been tested in various biological studies [16], and the extracts themselves or their compounds showed antioxidant [17,18,19,20,21,22,23,24], antiproliferative [17], estrogenic [25], antiatherosclerotic [26], anti-inflammatory [27], antibacterial [28], and antiviral activities. Japanese knotweed ethanolic extract inhibits the lytic cycle and reduces the production of Epstein –Barr (EBV) viral particles [29]. Ethanolic and water extracts (the latter at a higher dose) inhibit the production of hepatitis B virus (HBV) [30]. Methanolic extract inhibits infection with the mosquito-borne pathogen dengue virus (DENV) in the early viral entry phases and also reduces the infectivity of hepatitis C virus (HCV) and Zika virus (ZIKV) [31]. The antiviral effect of Japanese knotweed water extract was investigated in a model of acquired immunodeficiency syndrome in mice, and immunodeficiency was partially inhibited [32]. In addition, a 70% ethanolic_(aq)_ extract was tested against human immunodeficiency virus type 1 (HIV-1) and inhibited HIV-1-induced syncytium formation [33]. Bioactivity-guided fractionation enabled the isolation of 20 phenolic compounds with anti-HIV potential, and potent antiviral activity against HIV-1 was demonstrated for resveratrol, (+)-catechin, emodin-8-*O*-glucoside, and 5,7-dimethoxyphthalide [33]. Japanese knotweed water extract and its bioactive components resveratrol and emodin inhibit the replication of influenza A (H1N1) virus via interference with the mechanisms of the Toll-like receptor-9 pathway [34]. Furthermore, bioactivity-guided fractionation of the ethyl acetate extract was performed, and of the seven compounds isolated, resveratrol, (*E*)-3,5,12-trihydroxystilbene-3-*O*-beta-D-glucopyranoside-2′-(3″,4″,5″-trihydroxybenzoate) and catechin-3-*O*-gallate showed an inhibitory effect on neuraminidase activity, while the last two compounds also showed inhibitory activity against H1N1 [35].

Two important substances of Japanese knotweed rhizomes with well-studied and confirmed antiviral activity are emodin and resveratrol [36,37,38,39,40,41,42,43,44,45], phenolic compounds belonging to the anthraquinone and stilbene groups, respectively.

For emodin extracted from the rhizome of Japanese knotweed, antiviral activity against human simplex virus type 1 (HSV-1) was observed in guinea pigs [36]. Emodin inhibits the DNA replication of HBV [44]. Emodin and an ethyl acetate subfraction of Japanese knotweed rhizome, which contained 68.2% emodin, inhibited the expression of EBV immediate-early proteins and DNA replication [43]. Emodin isolated from Japanese knotweed inhibited the entry and replication of Coxsackie B4 virus (CVB_4_) and improved the survival of infected mice when administered orally [45].

Resveratrol inhibited the induced expression of early EBV antigen in Raji cells [41] and was proven useful in preventing the proliferation of EBV [37]. In addition, resveratrol inhibited the replication of human cytomegalovirus (HCMV) [38], varicella-zoster virus (VZV) [40], HSV-1 [39], and even HIV-1 [42].

Due to the beneficial effects of some traditional Chinese medicine (TCM) plants on SARS-CoV virus infection and other coronaviruses, these plants and their secondary metabolites have attracted attention and have been studied also within the current pandemic [46].

Emodin inhibited the interaction of SARS-CoV spike protein and ACE2 [47] and blocked coronavirus SARS-CoV and HCoV-OC43 ion channel 3a, which impaired virus release [48].

Resveratrol inhibited Middle East respiratory syndrome coronavirus (MERS-CoV) infection and prolonged cellular survival after infection. The expression of nucleocapsid protein essential for MERS-CoV replication decreased, and apoptosis induced by MERS-CoV in vitro was downregulated, after resveratrol treatment [49].

The aim of the current study was to test the antiviral activity of the 70% ethanol_(aq)_ extract of Japanese knotweed rhizome bark extract against live SARS-CoV-2 in vitro for the first time. A search in the literature shows a lack of in vitro studies as opposed to in silico studies to test the activity of this plant extract; the exceptions are Nawrot-Hadzik et al., 2021 [50], reporting the in vitro SARS-CoV-2 Mpro enzyme inhibitory activity of 70% acetone extract of the plant whole rhizome and Lin et al., 2022 [51], reporting the blocked entry of the SARS-CoV-2 pseudotyped virus into fibroblasts by water and ethanol extracts of the rhizome and root. The studies discussing Japanese knotweed or its compounds in connection with SARS-CoV are elaborated in detail in Results and Discussion.

## 2. Materials and Methods

### 2.1. Preparation of the Japanese Knotweed Rhizome Bark Extract

Rhizomes of Japanese knotweed were harvested in Ljubljana, Slovenia (Vrhovci, Mali Graben river bank; N 46°02′33.9″, E 14°27′00.9″). A voucher specimen was deposited in the Herbarium LJU (LJU10143477). The rhizomes were cleaned with tap water, the bark was peeled and lyophilized at −50 °C for 24 h (Micro Modulyo, IMAEdwards, Bologna, Italy), and the obtained dry material was frozen with liquid N_2_ and pulverized by a Mikro-Dismembrator S (Sartorius, Goettingen, Germany; 1 min, 1700 min^−1^). The rhizome bark powder (1 g) was extracted with 20 mL of 70% ethanol_(aq)_ (ethanol absolute anhydrous was purchased from Carlo Erba Reagents (Val de Reuil, France), and a Milli-Q water purification system (18 MΩ cm^−1^; Millipore, Bedford, MA, USA) was used to obtain ultrapure water). Vortexing (5 min), sonication (15 min) and centrifugation (5 min, 6700× *g*) were executed and the supernatant was transferred to a pre-weighted vial, while the extraction of the solid residue was repeated with 10 mL 70% ethanol_(aq)_. The solvent of the pooled supernatants was evaporated under N_2_ flow. The dry extract (414.17 mg) was dissolved in 127.37 mL of cell medium (extract stock solution) in a sterile hood and was used for antiviral activity assays without filtration.

### 2.2. Native SARS-CoV-2 Virus Neutralization Test (VNT)

Different concentrations of the extract from the stock solution (concentration 3.25 mg mL^−1^) in the culture media (ATCC; E-MEM with addition of 10% of FBS and 1% of standard antibiotic and antimycotic (Gibco, Grand Island, NY, USA; Anti-Anti 100x) were prepared in volume ratios of 1:64, 1:128, and 1:256. The number of infectious viral particles was quantified using the Median Tissue Culture Infectious Dose (TCID50) test. The assay is based on adding a serial dilution of the virus sample to the susceptible cells in a 96-well plate. The dilution at which 50% of the wells show CPE is used to mathematically calculate the TCID50 of the virus sample as generally described.

Extract dilutions (25 μL) and medium (25 μL) containing 6.2 TCID50 SARS-CoV-2 as virus working concentrations (SARS-CoV-2, 4265/20; EVAg) were mixed and incubated at 37 °C and 5% CO_2_ for 7 h. Each dilution of the extract and virus was incubated in 48 replicates in 96-well tissue culture plates (tissue culture test-plate 96F, TPP Techno Plastic Products AG, Trasadingen, Swiss). After the incubation period, 3.5 × 10^5^ Vero-E6 (African green monkey kidney cell line; ATCC CRL-1587) cell suspension was added in a volume of 100 μL to reach a final volume of 150 μL/well. To standardize the test procedures, a positive control with a working dilution of virus that was not incubated with the extract under the same conditions and volumes was included. After 110 h, the microplates were observed by inverted light microscope (ECLIPSE Ts2R, NIKON Instruments, Melville, NY, USA) at 400-fold magnification under a microscope equipped with an LED display. Characteristic morphological changes in the cells (rounding of adherent infected cells) in the culture that were the target of the SARS-CoV-2 replication were labelled as CPE. The other cellular transformation (cell dissolution and loss of cellular ultrastructure) was declared as a cytotoxic, necrotic effect. Each well in which the CPE was detected was labelled as positive for virus replication.

Before the main experiment, combinations of screening tests with different virus concentrations (virus working solution 100 TCID50 and subsequent dilutions in volume ratios of 1:2, 1:4, 1:8, 1:16, 1:32, 1:64, and 1:128) were performed in combination with different concentrations of extract (dilutions of stock solution in volume ratios of 1:2, 1:4, 1:8, 1:16, 1:32, 1:64, 1:128, 1:256, 1:512, 1:1024, and 1:2048) to determine the optimal concentration range for determining the cytotoxic properties of the extract and its potential antiviral activity under the same conditions as those under which the main experiment was later performed.

### 2.3. Statistical Analysis

The significance of the differences between the sums of the test and control groups in CPE incidence was determined using the one-way ANOVA test. A *p*-value of <0.05 was considered statistically significant. All the data were analyzed using Microsoft Excel 2016 (v16.0, Microsoft, Redmond, WA, USA).

## 3. Results and Discussion

A green solvent, 70% ethanol_(aq)_, suitable for tincture preparation was selected as an extraction medium for Japanese knotweed rhizome bark due to the obtained higher extraction yield (44.3%) in comparison to other solvents [52]. It is also considered less harmful when present as a residual solvent in pharmaceutical formulations compared to other organic solvents [53]. An additional advantage of ethanol is its commercial availability as a food-grade solvent. Japanese knotweed rhizome bark 70% ethanolic_(aq)_ extract was tested for its antioxidant activity in our previous study and was found to possess potent and stable time-dependent antioxidant activity [52].

In the search for an herbal antiviral drug and supported by already proven antiviral properties against other viruses, Japanese knotweed is one of several candidates to be tested against SARS-CoV-2. Within the current study we investigated the effect of the 70% ethanol_(aq)_ Japanese knotweed rhizome bark extract on the antiviral properties of a wild-type SARS-CoV-2 using the virus neutralization test (VNT), which is routinely used in laboratory diagnostics of viral diseases or in the detection of specific antibodies [54]. The test is also a suitable research tool for studying all potential antiviral substances, such as antiviral peptides, drugs, or disinfectants. The test detects prevention or inhibition of cytopathic effect (CPE) and viral replication if neutralizing antibodies or substances with antiviral properties are detected [54].

In the current study, the extract was incubated together with SARS-CoV-2 for 7 h before cell exposure.

It is illusory to expect a neutralizing effect in the range of the specific neutralizing antibodies (NAbs), making the partial reduction in the cytopathic effect in such an experimental model a realistic expectation.

One of the biological effects of NAbs is to reduce the infectivity of the virus by their specific binding to viral ligands, which block the attachment of the virus to the cell receptor, which is the principle of virus neutralization [54]. Other antiviral mechanisms include a wide spectrum of possibly undefined interactions at the molecular level between the virus and the host cell, leading to impaired virus infectivity or even to the destruction of the virus. The gold standard for evaluating the in vitro neutralization ability or antiviral properties of substances is the virus neutralization test with native, infectious virus, which most closely mimics the mechanism of virus–host interaction in natural infection. Accuracy is its key feature, requiring manipulation of wild-type live virus, and is therefore performed in level III biosafety standards laboratories. The closest alternative to this type of testing is the pseudovirus neutralization test, which uses a noninfectious pseudovirus and is therefore a safer test performed in level II laboratories, but the results must be interpreted with a certain degree of caution [54].

**Figure 1:**
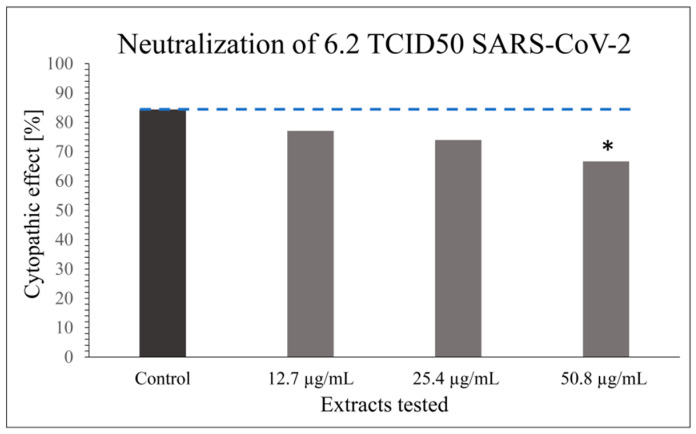
Graph showing the reduction in CPE by 70% ethanol_(aq)_ Japanese knotweed rhizome bark extract on SARS-CoV-2 performed in 48 parallels at extract concentrations that did not cause cytotoxicity in comparison with the optimal working concentration of the virus (6.2 TCID50 SARS-CoV-2). The results are presented as a percentage of wells with cytopathic effect on Vero-E6 cells (*n* = 48); * *p* < 0.05. The result for the positive control (cells exposed to the virus only) is presented for comparison.

**Figure 2:**
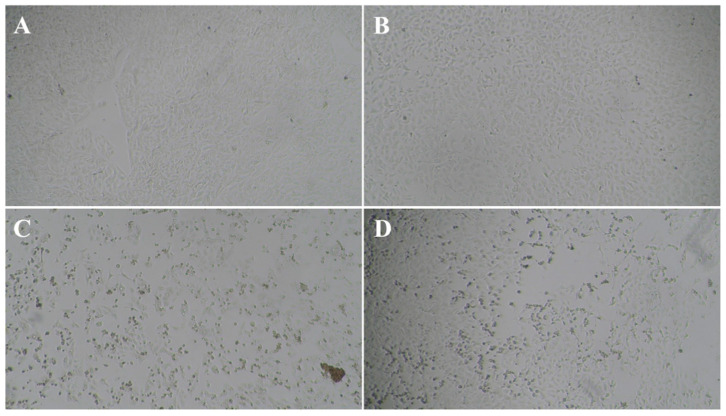
Images of: (**A**) non-treated Vero-E6 cells; (**B**) Vero-E6 cells exposed to SARS-CoV-2 virus (6.2 TCID50 SARS-CoV-2) and Japanese knotweed rhizome bark extract in concentration which prevents a cytopathic effect without provoking cytotoxicity (50.8 µg mL^−1^); (**C**) cytotoxic effect caused by higher concentrations of Japanese knotweed rhizome bark extract; (**D**) cytopathic effect resulting from viral infection.

Modifications of VNT have been described in the scientific literature to investigate the antiviral properties of such herbal extracts [51]. However, only laboratory testing models with a wild-type virus can most suitably mimic the natural viral infection of the host by allowing long-term virus–cell contact with prolonged antiviral contact of the virus extract in the experiment. The obtained results indicate that the tested extract has a certain level of inhibitory properties on the viral infectivity cycle; however, these are not as potent as the specific neutralizing antibody. CPE or antiviral effect of SARS-CoV-2 was studied in 48 parallels at each virus’ and extract’s concentration, taking into consideration only dilutions of the extract stock solution that did not cause cytotoxicity (1:64; 1:128; 1:256; *v/v*) in combination with the optimal working concentration of the virus (6.2 TCID50 SARS-CoV-2) (Figure 1 and Figure 2) as previously determined by screening tests.

A statistically significant antiviral effect (reduction in CPE) against SARS-CoV-2 was observed for the stock solution diluted 1:64 (*v/v*) (i.e., 50.8 µg mL^−1^) compared to the incidence of CPE in the positive control, where the virus-working concentration was inoculated on Vero-E6 cells only; *p* < 0.05 (Figure 1). Besides the statistically significant result for the CPE reduction effect of the extract at 50.8 µg mL^−1^ compared to the positive control, a dose–response relationship was generally noticed for all the extract concentrations (Figure 1).

The interactions between Japanese knotweed flavan-3-ols and proanthocyanidins [52,55] and FBS albumin in the cell medium should be considered when evaluating Japanese knotweed extract using in vitro cell culture systems. Namely, among various biological activities, Japanese knotweed polyphenols possess astringent activity [56]. The VNT executed with the filtered extract dissolved in cell medium did not show promising results. Therefore, final experiments were performed with the non-filtered extract to avoid possible removal of these complexes. According to the literature, interactions between flavan-3-ols and FBS albumin may induce cytotoxicity at high concentrations of flavan-3-ols [57]. In the current study, the cytotoxic effect was observed at extract concentrations above 50.8 µg mL^−1^. Although SARS-CoV-2 inhibitory activity was expected to be even higher at higher concentrations of the extract, this could not be evaluated at cytotoxic concentrations.

The mechanisms of antiviral activity on SARS-CoV-2 inhibitory activity and the main antiviral compounds of the examined extract as well as possible cell protection effect should be investigated on a molecular level in a further study. To support the obtained result, a few studies elaborating the effect of Japanese knotweed extract and its compounds on SARS-CoV virus are described below; however, none of these studies involve live, wild-type SARS-CoV-2 virus.

An herbal drug called Shufeng Jiedu, consisting of eight medicinal plants including Japanese knotweed, is known for its antiviral, anti-inflammatory, and immunomodulatory effects in acute lung diseases and was discussed as a promising candidate for the treatment of COVID-19 [58]. However, the antiviral and anti-inflammatory effects of Shufeng Jiedu were tested in a HCoV-229E (group 1 coronavirus) mouse model [58]. The formulation reduced viral load and decreased inflammatory factors in the lungs while increasing the concentration of CD4^+^ and CD8^+^ cells in the blood [58]. Direct binding of polydatin (an anthraquinone from the Japanese knotweed rhizome [52,55]), quercetin, and wogonin to the major protease of SARS-CoV-2 was demonstrated in silico [58]. Clinical data for Shufeng Jiedu added to standard antiviral therapy showed significant reduction in the COVID-19 clinical recovery time. Still, largescale, randomized, placebo-controlled, double-blinded clinical trials are lacking.

In addition, Baidu Jieduan granules containing Japanese knotweed and eleven other herbs have been clinically evaluated for efficacy and safety in the treatment of moderate COVID-19 [59]. Nonetheless, placebo-controlled and double-blinded clinical trial design is lacking.

The results of network pharmacology and bioinformatics analysis suggest that Japanese knotweed material is a promising therapeutic agent against COVID-19 [60].

The antiviral activity of purchased standards of polydatin and resveratrol, typical compounds of Japanese knotweed, has been demonstrated in vitro against HCoV-OC43 strain, an alternative model for SARS-CoV-2 [61]. Furthermore, computer-aided virtual screening was used to predict the binding site, and surface plasmon resonance (SPR) analysis was employed to confirm the interaction. SPR results showed a specific affinity of polydatin and resveratrol toward SARS-CoV 3CLpro and PLpro proteins as well as toward SARS-CoV-2. Moreover, Japanese knotweed was suggested as a potential therapeutic agent for pulmonary fibrosis caused by COVID-19 based on network pharmacology and data mining [62].

In other studies, a decreased infectivity of the SARS-CoV-2 spike pseudovirus in HEK293T-ACE2 cells was observed by polydatin [63], while resveratrol-inhibited SARS-CoV-2 replication (0–99.3%) in a dose-dependent manner (0–25 µM) with an EC90 and EC50 (50% and 90% maximal effective concentration, respectively) 11.42 and 10.66 µM, respectively. Higher concentrations of resveratrol (>50 µM) posed cytotoxicity on Vero-E6 cells [64].

In the study by Nawrot-Hadzik et al., 2021 [50], 25 compounds known to be present in the rhizomes of *Reynoutria japonica* (Japanese knotweed) and *Reynoutria sachalinensis* were docked into the main protease binding site of SARS-CoV-2. Further, 11 of them together with the extracts of both plants were tested in vitro for inhibition of SARS-CoV-2 Mpro enzyme [50]. Vanicosides A and B, isolated from the rhizomes of *Reynoutria sachalinensis*, showed moderate inhibitory activity against SARS-CoV-2 Mpro, while acetone extract and especially the butanol fractions of plants (of tested: dichloromethane, diethyl ether, ethyl acetate, *n*-butanol, and water fractions) containing vanicosides and polymerized procyanidins showed strong inhibition against SARS-CoV-2 Mpro [50]. Water and 90% ethanol_(aq)_ extracts of Japanese knotweed rhizome and root blocked the entry of SARS-CoV-2 pseudotyped virus into HEK293T-ACE2 cells and zebrafish larvae and were shown to inhibit the spike protein–ACE2 receptor interaction and 3CL protease activity. Anti-SARS-CoV-2 activity was confirmed for the extract component gallic acid [51].

Some doubts and counter-opinions about the antiviral activity of Japanese knotweed against SARS-CoV-2 are also present in the literature. For instance, although the frequency of COVID-19 infection cases should be lower in Asian countries where Japanese knotweed occurs as a component of local foods, this has not been the case [65].

The results of the current study could be prospectively used to formulate or at least promote food supplements containing a Japanese knotweed rhizome bark extract for the prevention or complementary treatment of COVID-19 patients. A biofoil enriched with this extract which could be used as active packaging for food, drugs, and cosmetics to protect food or other packed contents from bacteria and oxidation has already been formulated by our group [66]. These foils may potentially prevent the spread of coronavirus (or other virus species), since another possible way for coronavirus to spread, besides through respiratory air (as mentioned in the introduction) is via indirect contact with contaminated surfaces. The virus remains viable and infectious in aerosols for hours and on surfaces for up to a day [67]. It is particularly stable on conventional plastic and stainless steel [67]. Other hygiene products such as soaps (soap destabilizes the lipid bilayer of viruses [68]) and disinfectant solutions (ethanol or isopropanol inactivate human coronavirus [69]) could also be enriched with Japanese knotweed rhizome bark extract to enhance their effect (act as bio-disinfectants). In this way, we would fight two battles at the same time: potential new COVID-19 waves and the invasiveness of Japanese knotweed, as the best way to eradicate or at least restrict this plant from Europe and North America (where it represents a major economic and environmental problem) is via mechanical excavation, which, on the other hand, is a necessary step to obtain extracts from its rhizome bark.

## Data Availability

All the data generated for this study are included in the article.
